# Persistent Hypoxia with Intermittent Aggravation Causes Imbalance in Smad3/Myocardin-Related Transcription Factor Signaling with Consequent Endothelial Senescence and Pulmonary Arterial Remodeling

**DOI:** 10.3390/biomedicines11092351

**Published:** 2023-08-23

**Authors:** Jiaxin Hu, Prachi Singh, Jingrui Li, Jing Zhang, Fei Li, Hehe Zhang, Jiang Xie

**Affiliations:** 1 Department of Respiratory and Critical Medicine, Beijing Anzhen Hospital, Capital Medical University, Beijing 100029, China; hujiaxin@mail.ccmu.edu.cn (J.H.); lftutu@163.com (F.L.); zhanghehehe2023@126.com (H.Z.); 2 Pennington Biomedical Research Center, Baton Rouge, LA 70808, USA; prachi.singh@pbrc.edu; 3 First Hospital of Lanzhou University, Lanzhou 730009, China; jrli2020@lzu.edu.cn; 4 Beijing Institute of Heart, Lung and Blood Vessel Diseases, Beijing 100029, China; zhangj_1229@163.com; 5 Beijing Anzhen Hospital Centre for Sleep Medicine and Science, Capital Medical University, Beijing 100029, China

**Keywords:** myocardin-related transcription factor, PI hypoxia, remodeling, senescence, Smad3

## Abstract

Loss of Smad3 and the consequent activation of myocardin-related transcription factor (MRTF) are associated with vascular pathologies. This study aimed to examine the impact of persistent hypoxia with intermittent aggravation (PI hypoxia) on cellular senescence and pulmonary arterial remodeling mediated by the Smad3/MRTF imbalance. We examined the effects of PI hypoxia on the Smad3/MRTF pathway and cellular senescence using human pulmonary artery endothelial cells (HPAECs) and in vivo studies in rats. The senescent degree was evaluated using β-galactosidase staining, p16 quantitation and the measurement of senescence-associated secretory phenotype. Structural data in the pathological analysis of pulmonary artery remodeling were collected. Compared to the control, HPAECs and pulmonary tissue from rats exposed to PI hypoxia showed a significantly higher senescent degree, lower expression of Smad3, and higher MRTF levels. The overexpression of Smad3 significantly mitigated HPAECs senescence in vitro. Further, treatment with CCG-203971, which inhibits MRTF, increased Smad3 levels and reduced β-galactosidase positive cells in rat lung tissue. This intervention also alleviated PI hypoxia-induced pathological changes, including remodeling indices of pulmonary arterial thickening, muscularization, and collagen formation. In conclusion, imbalanced Smad3/MRTF signaling is linked to PI hypoxia-induced senescence and pulmonary arterial remodeling, making it a potential therapeutic target for patients with sleep apnea and chronic obstructive pulmonary disease.

## 1. Introduction

Overlap syndrome (OS) is characterized by the co-occurrence of obstructive sleep apnea (OSA) and chronic obstructive pulmonary disease (COPD). The overall prevalence of OSA-COPD OS ranges from 1 to 3.6% in the general population [1], whereas in patients with moderate-to-severe COPD, the prevalence of OS has been reported to be as high as 65.9% [2]. Patients with advanced OS may present with persistent hypoxia due to impaired ventilatory capacity and intermittent hypoxia (IH) caused by recurrent obstruction of the upper airway during sleep. This leads to a unique form of hypoxia, referred to as persistent hypoxia with intermittent aggravation (PI hypoxia), which generates significant hypoxaemia. Studies have suggested that PI hypoxia is closely linked to the development of pulmonary arterial diseases [3,4]. To better understand the pathophysiology of pulmonary hypertension and develop more effective treatments, it is crucial to explore the mechanisms underlying pulmonary remodeling caused by PI hypoxia. Despite the widespread use of current therapeutic strategies for pulmonary hypertension, including endothelin receptor antagonists and phosphodiesterase type 5 inhibitors, a considerable proportion of patients experience disease progression. This highlights the need for alternative approaches and novel targets to enhance treatment efficacy and improve patient outcomes.

Senescence is a biological process in which cellular functions deteriorate, resulting in a decline in cellular proliferation and differentiation. The dynamic balance between senescence and rejuvenation is critical for maintaining the health of an organism. However, excessive senescence is associated with pathological impairments such as atherosclerosis [5]. Studies in both animals and humans have shown that the senescence of pulmonary artery endothelial cells is strongly connected to the development of irreversible pulmonary hypertension [6,7]. Continuous hypoxia and chronic IH, both of which are crucial factors in mediating pulmonary arterial damage, have been demonstrated to induce senescence [8,9]. For example, our previous study revealed that IH, which mimics the hypoxic state of OSA, induced a high expression of senescence-associated β-galactosidase (SA-β-gal) in human adipose precursor cells [8]. Currently, there is a lack of research on the relationship between PI hypoxia and respiratory senescence, as well as its role in the structural impairment of the pulmonary artery.

Smads (small body size [aC. elegans protein] mothers against decapentaplegic [a Drosophila protein family]) are a class of vital proteins that transmit transforming growth factor-β signals from cell surface receptors into the nucleus. However, unphosphorylated Smad3 can also enter the nucleus to exert biological effects [10]. Furthermore, intracellular Smad3 protein not only causes tissue damage via the classical transmit transforming growth factor-β/Smads pathway but also exerts protective effects on cellular growth, differentiation, and immune regulation [11,12]. Functionally, Smad3 plays an indispensable role in protecting the vascular system [12,13,14], likely because it inhibits the activation of the myocardin-related transcription factor (MRTF) [13], a mediator of vascular injuries. In monocrotaline or hypoxia-induced models, Smad3 expression is reduced in the pulmonary arteries, along with its decreased inhibitory effect on MRTF [13]. Moreover, in hypoxia-exposed rats, the inhibition of MRTF has been shown to attenuate vascular remodeling [13], a significant pathological basis for the development of pulmonary hypertension. To our knowledge, no studies have investigated the impact of Smad3/MRTF imbalance on the senescence of pulmonary vascular cells. Given the involvement of Smad3 and MRTF in hypoxia-induced pulmonary artery injury, we aim to explore the relationship between Smad3/MRTF imbalance and the degree of senescence in the respiratory system. This investigation could provide valuable insights into the pathogenesis of pulmonary arterial remodeling and identify potential therapeutic targets. Therefore, we constructed PI hypoxia models to verify our hypothesis that the loss of Smad3 and unleashed MRTF mediate senescence and pulmonary arterial remodeling, which can be targeted for alleviation.

## 2. Materials and Methods

### 2.1. Cell Culture and Treatment

Human pulmonary artery endothelial cells (HPAECs) (Beina Chuanglian Biotechnology Research Institute, Beijing, China) were cultured in 1640 medium (Biological Industries, Israel) with 10% fetal bovine serum (Gibco, Billings, MT, USA) for 72 h in a cell culture incubator (SANYO, Osaka, Japan), which provided a continuous supply of 5% carbon dioxide. Changes in the oxygen concentration (FiO_2_) in the incubator were achieved by filling it with nitrogen (Thermo Fisher Scientific, Waltham, MA, USA).

The PI hypoxia model was designed based on previous cell models of IH [15] and persistent hypoxia [16]. To achieve the hypoxic conditions required for the cellular study, the cells were subjected to cyclically alternating 4 h of PI hypoxia with 4 h of continuous hypoxia. The PI hypoxia program was administered as follows: first, the FiO_2_ in the incubator was rapidly reduced from 21% to 15% to establish the basic environment. Then, hypoxia aggravation (60 events/h) was initiated to reduce FiO_2_ from 15% to 5% within 30 s, followed by restoring FiO_2_ from 5% to 15% in the next 30 s. The FiO_2_ of 15% was maintained during the interval of hypoxia aggravation. All the above processes, including normoxia, continuous hypoxia, and intermittent aggravation of hypoxia, were controlled using a preset computerized program.

The goal of these in vitro studies was to (1) examine the effects of PI hypoxia and (2) evaluate the role of Smad3/MRTF in mediating the effects of PI hypoxia. Therefore, four subgroups were created for all the outcomes: (1) normoxia (control), (2) PI hypoxia (PI), (3) Smad3 overexpression in PI hypoxia (PI + Smad3-OE), and (4) CCG-203971 (MRTF inhibitor, 5 mmol/L, Selleck Chemicals; Houston, TX, USA) treatment in PI hypoxia (PI + CCG-203971). The Smad3 overexpression was achieved using a specific lentivirus construct as described below [17].

### 2.2. Animals

Male rats aged 6–8 w were purchased from Weitong Lihua and were raised in a hypoxic chamber (AIPUINS Instruments; Hangzhou; China). The rats were provided with normal chow and were kept in a 12–12 h light–dark cycle environment with a temperature of 20–24 °C and a humidity of 50–60%. After 2 d of adaptive feeding with normoxia, the rats were randomly divided into 3 subgroups: (1) normoxia, (2) PI hypoxia (PI), and (3) CCG-203971 treatment in PI hypoxia (PI + CCG-203971). The last group was given daily intraperitoneal injections of CCG-203971 (0.15 mg/kg) during PI hypoxia feeding, while the other groups were injected with an equal volume of 0.9% sodium chloride. Approval for this animal study (ks2019020) was obtained from the Institutional Review Board of Beijing Anzhen Hospital.

The rats were initially raised in a normoxic environment for 2 d before being transferred to a hypoxic chamber for 21 d. The FiO_2_ was set to 15 ± 1% for continuous hypoxia and 8–15% for IH aggravation. Since the maximum FiO_2_ was not higher than 15% during both IH and persistent hypoxia, the rats were in PI hypoxia. The concentration of CO_2_ in the hypoxic chamber was maintained at normal levels by absorbing excessive CO_2_ using calcium oxide and calcium hydroxide. The chamber was opened every 3 d at 12 pm for cleaning and to supply food and water, with each opening not exceeding 30 min. The circadian rhythm of daytime and nighttime alternated every 12 h. Moderate or severe hypoxaemia was confirmed via arterial blood gas analysis. Rats in the control group were placed in an air environment with the same temperature and humidity.

The PI hypoxia strategies in animal studies were as follows: the chamber was filled with nitrogen to control the FiO_2_ at 15 ± 1% to maintain continuous hypoxia from 9 pm to 9 a.m., while IH aggregation was implemented from 9 a.m. to 9 p.m. In each cycle of hypoxia aggravation, the FiO_2_ in the chamber was decreased to 8% in 80 s and gradually recovered to 15% in 20 s. FiO_2_ of 15% was maintained for 20 s until the beginning of the next cycle of hypoxia aggravation. The main control board regulated the conversion of nitrogen and compressed air in the cabin to maintain a simulated OSA rate of 30 events/h on the basis of moderate-to-severe COPD.

### 2.3. Smad3 Overexpression

Lentivirus overexpressing Smad3 was purchased from Yi Berry (Shanghai, China). HPAECs were seeded into six-well plates at a density of 5 × 10^4^ per well and then transfected with lentivirus overexpressing Smad3 using Lipofectamine 3000 (Invitrogen, Carlsbad, CA, USA). The cells were first cultured using a complete medium for 72 h and then replaced with a medium containing puromycin (0.5 μg/mL) to screen the HPAECs stably expressing Smad3 for 7 d. The transfection efficiency was examined via quantitative real-time polymerase chain reaction (qRT-PCR), which is shown in Supplementary File S1: Figure S1.

### 2.4. Senescence Assessed by SA-β-gal

SA-β-gal was one of the most commonly used methods to assess cellular or tissue senescence and adapted from previously published protocols [18,19,20]. Briefly, the senescent status of HPAECs and lung tissue cells was determined using an SA-β-gal staining kit (Cell Signaling Technology, Danvers, MA, USA). SA-β-gal staining fixative was added to each 2 × 10^4^ cell or lung tissue slide for 15 min at room temperature and washed with phosphate buffer solution. Then, SA-β-gal staining solution was added to the cells or lung tissue slides and incubated overnight at 37 °C. After removing the SA-β-gal staining solution, the cells were covered with 70% glycerin gelatine and incubated at 4 °C. Senescent cells that appeared in blue were observed using a microscope (Nikon, Tokyo, Japan). We counted the number of stained cells (blue staining in vitro and in vivo) per 10^6^ um^2^ to assess cell senescence.

### 2.5. Flow Cytometry Assay for Cell Apoptosis

The Annexin V-APC/PI apoptosis detection kit (Southern Biotech, Birmingham, AL, USA) was used to assess the cellular apoptosis according to the manufacturer’s instructions. HPAECs were digested by trypsinization, centrifuged at 1300 rpm for 3 min and washed with phosphate-buffered solution. The cells were suspended in an annexin V binding buffer. An APC-labeled Annexin V and propidium iodide were added to the cells, followed by 15 min incubation in the dark. The mixture was centrifuged at 1500 rpm for 3 min to remove the supernatant and 1000 μL diluted 1 × binding buffer was added. The cells were tested via MM high-pass flow cytometer (Millipore, Burlington, MA, USA), with a percentage of apoptotic cells analyzed using FlowJo software version 10.8.1 (Treestar, Ashland, OR, USA).

### 2.6. Histopathological and Immunohistochemistry Staining

For the histopathological analysis, the lung tissue from rats was fixed in 4% paraformaldehyde, processed and embedded in paraffin, cut into 4 µm sections, and stained with hematoxylin and eosin (H&E) or Masson trichrome (Baso, Zhuhai, China). For immunohistochemistry of the smooth muscle proliferation, the lung tissue sections were subjected to induced antigen retrieval and subsequently labelled with the antibody of anti-alpha-smooth muscle actin (α-SMA) protein at 4 °C overnight and then incubated with HRP-conjugated secondary antibody. The sections were captured using a microscope (Olympus, Tokyo, Japan), and visualized via the CaseViewer (version 2.4, 3DHISTECH, Budapest, Hungary) software to select pulmonary arterioles with a blood vessel diameter of 80–150 μm. The results were analyzed using Image J software version 1.53 [21].

### 2.7. Right Ventricular Hypotrophy Index (RVHI)

For determining the RVHI, the cardiac atrium was carefully excised, followed by the separation of the right ventricle (RV), left ventricle and interventricular septum (LV + S) along the ventricular septum. The RV and LV + S were weighed and RVHI was calculated using the formula RVHI = RV/(LV + S) [22].

### 2.8. Further Applied Methods

Additional qRT-PCR, Western Blot, enzyme-linked immunosorbent assay (ELISA) and cell counting kit-8 proliferation assay (CCK8) are further described in Supplementary File S2.

### 2.9. Statistical Analysis

All experiments were performed with at least three independent biological experiments. Continuous data were presented as mean and standard deviation. Student’s *t*-test or one-way ANOVA was used to compare whether there were statistical differences between two or more groups, and Bonferroni or Dunnet method was used for post hoc testing. Pearson correlation analysis was conducted to measure the strength and direction of the relationship between two continuous variables. Statistical analysis was conducted using JMP software, Version 16.1 (SAS Institute, Cary, NC, USA) and GraphPad software, Version 9.0 (San Diego, CA, USA) with a significance level of two-sided *p* < 0.05.

## 3. Results

### 3.1. Smad3 Loss Mediates Senescence Induced by PI Hypoxia

**Figure 1:**
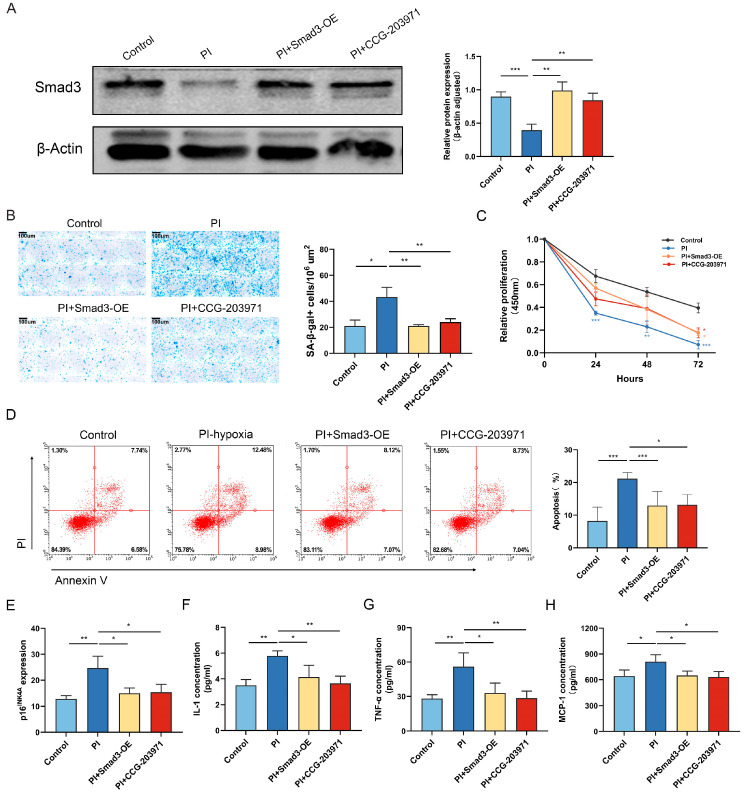
Overexpression of Smad3 mitigates HPAECs senescence and apoptosis induced via PI hypoxia in vitro. (**A**) Representative Western Blot and graph of the relative protein levels of Smad3 in HPAECs. (**B**) Representative image and statistical result of SA-β-gal+ (blue staining) HPAECs abundance per area in each group. (**C**) CCK-8 results for the ability of proliferation in HPAECs. (**D**) Representative image of flow cytometry and statistical result for the apoptotic HPAECs and percentages. (**E**) qPCR analysis of p16^INK4A^ gene expression. ELISA analysis of SASP factor concentration of (**F**) IL-1, (**G**) TNF-α, and (**H**) MCP1. Data presented are mean ± SD of three independent experiments. * *p* < 0.05; ** *p* < 0.01; *** *p* < 0.001.

Compared to normoxia, 72 h PI hypoxia exposure in cultured HPAECs decreased Smad3 expression (Figure 1A) and increased the presence of senescent cells (Figure 1B). The consequent decrease in cellular proliferation (Figure 1C) and increase in apoptosis (Figure 1D) were also observed with PI hypoxia treatment. The expression of p16^INK4a^ (Figure 1E) and senescence-associated secretory phenotypes (SASP) factor concentration of interleukin-1 (IL-1), tumor necrosis factor alpha (TNF-α), and monocyte chemoattractant protein-1 (MCP1) (Figure 1F–H) were increased after PI hypoxia. Notably, the detrimental effects of PI hypoxia on HPAECs were reversed with Smad3 overexpression.

**Figure 2:**
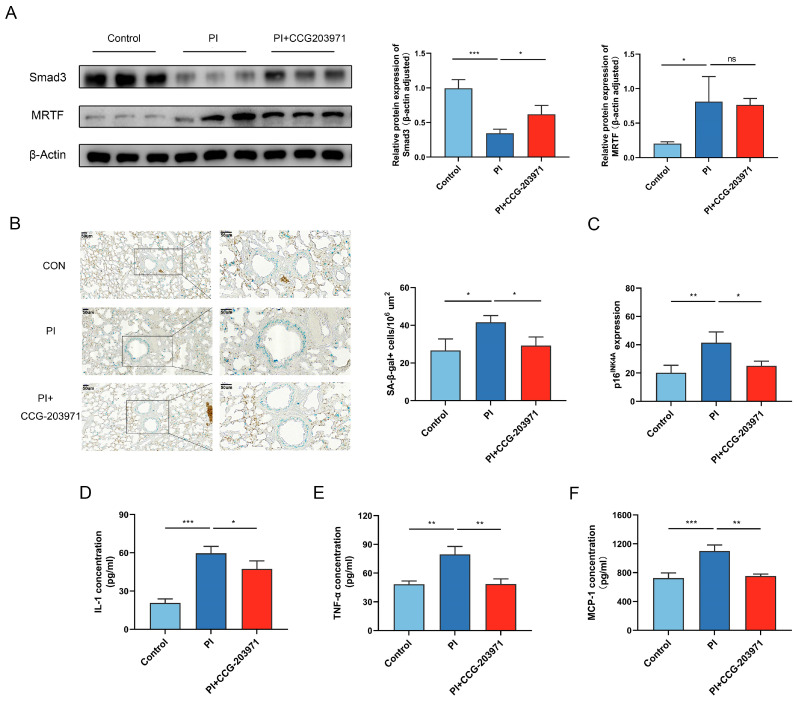
PI hypoxia induces senescence in vivo, which is mitigated via MRTF inhibition. (**A**) Representative Western Blot and graph of the relative protein levels of Smad3 and MRTF in tissue. Histological image and the statistical result of (**B**) SA-β-gal+ (blue staining) cells abundance per area in each group. (**C**) qPCR analysis of p16^INK4A^ gene expression. ELISA analysis of SASP factor concentration of (**D**) IL-1, (**E**) TNF-α, and (**F**) MCP1. Data presented are mean ± SD of three independent experiments. * *p* < 0.05; ** *p* < 0.01; *** *p* < 0.001. ns: not significant

Similar to our in vitro findings, rats exposed to PI hypoxia for 21 d showed significant Smad3 loss in their lung tissue (Figure 2A). Senescence assessed via SA-β-gal staining was seen in the lung tissue of rats exposed to PI hypoxia (Figure 2B), and p16^INK4A^ gene expression and SASP factor concentration of IL-1, TNF-α, and MCP1were confirmed to be upregulated in the peripheral blood of rats after PI hypoxia exposure (Figure 2C–F).

### 3.2. PI Hypoxia Activates MRTF, Causing Senescence and Pulmonary Arterial Remodeling

**Figure 3:**
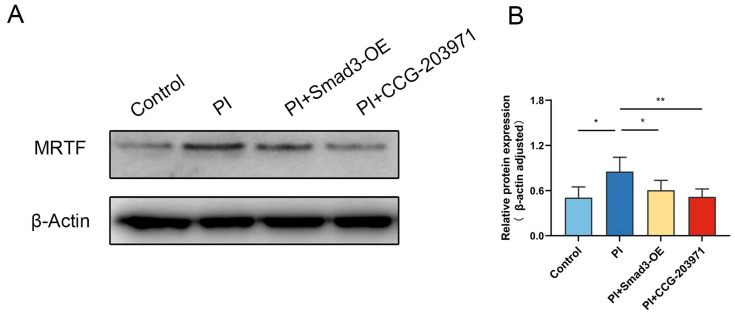
MRTF expression is upregulated in HPAECs after PI hypoxia in vitro. (**A**) Representative Western Blot of MRTF in HPAECs after PI hypoxia exposure. (**B**) Quantification for relative protein levels of MRTF via densitometric analysis in each group. Data presented are mean ± SD of three independent experiments. * *p* < 0.05; ** *p* < 0.01.

Smad3 is known to regulate the expression of MRTF [23]; therefore, we next evaluated the effects of PI-hypoxia on MRTF expression and activity. The expression of MRTF was higher in the PI group than in the control group, which was reduced via MRTF inhibitor (CCG-203971) treatment and Smad3 overexpression (Figure 3A,B). The administration of CCG-203971 decreased cell counts with positive SA-β-gal staining (Figure 1B). Similarly, the treatment of CCG-203971 attenuated the increase of p16^INK4A^ gene expression and SASP factor concentration of IL-1, TNF-α, and MCP1 induced via PI hypoxia (Figure 1E–H), suggesting the ability of MRTF inhibitor to curtail senescence in cultured HPAECs exposed to 72 h PI hypoxia. Meanwhile, the PI + CCG-203971 group exhibited significantly improved HPAEC proliferation (Figure 1C) and a reduced proportion of apoptotic cells (Figure 1D) than the PI group.

**Figure 4:**
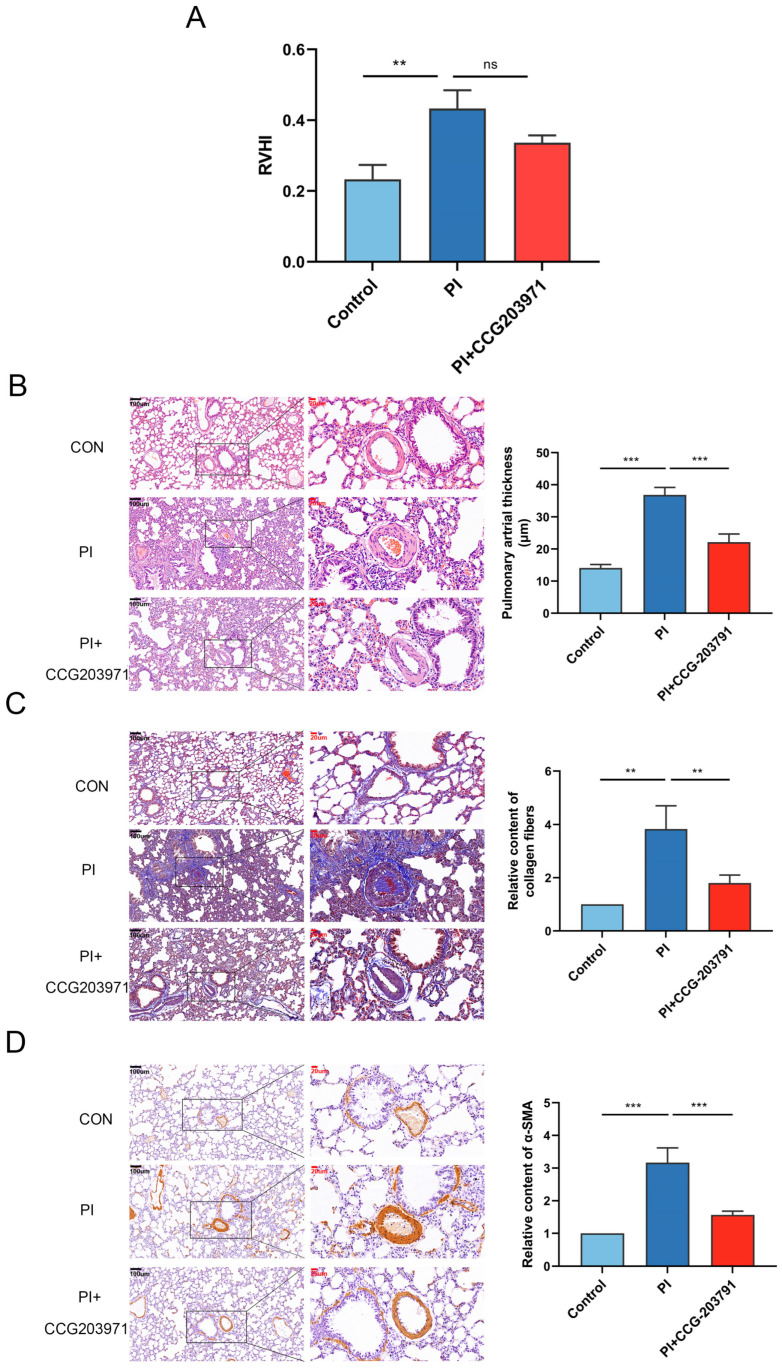
PI hypoxia induces pulmonary artery remodeling in vivo, which is mitigated via MRTF inhibition. (**A**) RVHI of each group using the anatomical method. Histological image and statistical result of (**B**) thickness of pulmonary arterial vessel wall in lung tissue via H&E staining, (**C**) relative level of collagen fiber via Masson staining, and (**D**) proliferation of smooth muscle via relative expression for α-SMA by IHC. Data presented are mean ± SD of three independent experiments. ** *p* < 0.01; *** *p* < 0.001. ns: not significant

Compared with the control group, MRTF expression in the lung tissue of PI hypoxia animals was significantly higher (Figure 2A). MRTF was slightly inhibited along with significant restoration of Smad3 expression when therapeutic CCG-203971 was administered. Meanwhile, CCG-203971 treatment could not only mitigate PI hypoxia-induced senescence (evaluated via SA-β-gal staining) (Figure 2B), but also downregulated the level of p16^INK4A^ gene expression and SASP factor concentration of IL-1, TNF-α, and MCP1 (Figure 2B–E). There was no significant improvement in the value of RVHI (Figure 4A). As the pathological results showed, treatment with CCG-203971 resulted in a structural improvement of PI hypoxia-induced damage, such as thinner pulmonary arterioles wall (Figure 4B), reduced deposition of collagen fibers (Figure 4C), and decreased muscularization assessed via α-SMA expression (Figure 4D) in the PI + CCG-203971 group as compared with the PI group.

### 3.3. Smad3/MRTF Imbalance Associates with Senescence and Vascular Remodeling

**Figure 5:**
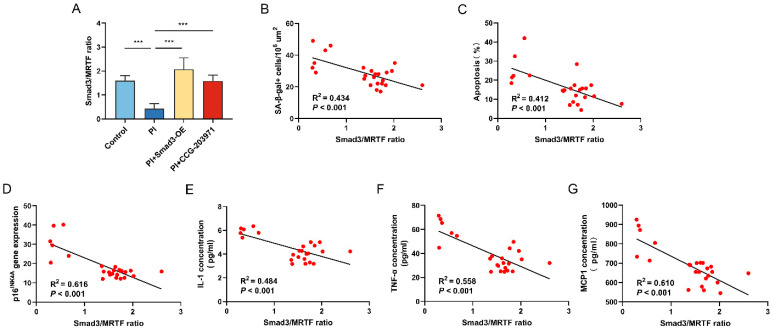
Smad3/MRTF imbalance is associated with cellular senescence after PI hypoxia exposure in vitro. (**A**) The ratio of Smad3/MRTF was calculated by dividing the protein level of Smad3 by the protein level of MRTF according to the results of Western Blot in HPAECs. The correlation analysis of Smad3/MRTF ratio with (**B**) SA-β-gal+ cell abundance per area, (**C**) percentage of apoptotic HPAECs, (**D**) p16^INK4A^ gene expression, and SASP factor concentration of (**E**) IL-1, (**F**) TNF-α, and (**G**) MCP1 in HPAECs. Data presented are mean ± SD of three independent experiments. *** *p* < 0.001.

The ratio of Smad3/MRTF was calculated by dividing the protein level of Smad3 by the protein level of MRTF. The results showed that the ratio was lower in HPAECs cultured in PI hypoxia than in normoxia, which can be reversed, respectively, either by Smad3 overexpression or by CCG-203971 (Figure 5A). Furthermore, the results of correlation analysis showed that the value of Smad3/MRTF was negatively correlated with the level of SA-β-gal (R^2^ = 0.434, *p* < 0.001), cellular apoptosis (R^2^ = 0.412, *p* < 0.001), p16^INK4A^ gene expression (R^2^ = 0.616, *p* < 0.001) and SASP of IL-1 (R^2^ = 0.484, *p* < 0.001), TNF-α (R^2^ = 0.558, *p* < 0.001), and MCP1 (R^2^ = 0.610, *p* < 0.001) (Figure 5B–G).

**Figure 6:**
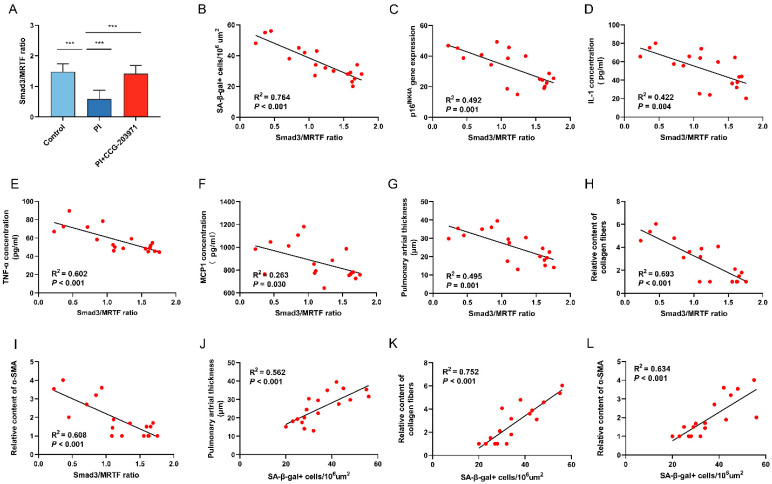
Smad3/MRTF imbalance is associated with senescence and pulmonary artery remodeling after PI hypoxia exposure in vivo. (**A**) The ratio of Smad3/MRTF was calculated by dividing the protein level of Smad3 by the protein level of MRTF according to the results of Western Blot in lung tissue. Correlation analysis of Smad3/MRTF ratio with (**B**) abundance of SA-β-gal+ cells in lung tissue, (**C**) p16^INK4A^ gene expression, and SASP factor concentration of (**D**) IL-1, (**E**) TNF-α and (**F**) MCP1 in lung tissue. Correlation analysis of Smad3/MRTF ratio with (**G**) thickness of pulmonary arterial vessels in lung tissue, (**H**) relative level of collagen fiber and (**I**) α-SMA expression. Further correlation analysis of SA-β-gal level with (**J**) thickness of pulmonary arterial in lung tissue, (**K**) collagen fiber and (**L**) α-SMA expression. Data presented are mean ± SD of three independent experiments. *** *p* < 0.001.

Similar to the in vitro study, Smad3/MRTF ratio was lower in the lung tissue of rats exposed to 21 d of PI hypoxia than in normoxia, which was substantially regained by CCG-203971 treatment (Figure 6A). A negative correlation existed between the value of Smad3/MRTF and SA-β-gal expression (R^2^ = 0.764, *p* < 0.001) (Figure 6B). Smad3/MRTF was also negatively correlated with p16^INK4A^ gene expression (R^2^ = 0.492, *p* = 0.001) and SASP of IL-1 (R^2^ = 0.422, *p* = 0.004), TNF-α (R^2^ = 0.602, *p* < 0.001) and MCP1(R^2^ = 0.263, *p* = 0.030) (Figure 6C–F). Further, Smad3/MRTF was found to be negatively correlated with the remodeling indices of pulmonary arterial thickness (R^2^ = 0.495, *p* = 0.001), collagen fiber amount (R^2^ = 0.693, *p* < 0.001) and α-SMA expression (R^2^ = 0.608, *p* < 0.001) (Figure 6G–I). Additionally, we found that SA-β-gal expression was positively correlated with the remodeling indices of pulmonary arterial thickness (R^2^ = 0.562, *p* < 0.001), collagen fiber amount (R^2^ = 0.752, *p* = 0.002) and α-SMA expression (R^2^ = 0.634, *p* < 0.001) (Figure 6J–L).

### 3.4. PI Hypoxia Extends Telomere Length (TL)

**Figure 7:**
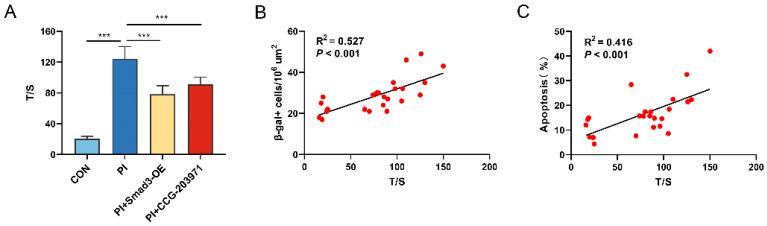
TL is prolonged and associated with senescence after PI hypoxia exposure in vitro. (**A**) TL evaluated as the ratio of T/S according to the results of qRT-PCR in HPAECs. Correlation analysis of T/S ratio with (**B**) abundance of SA β-Gal+ HPAECs, and (**C**) percentage of apoptotic cells. Data presented are mean ± SD of the three independent experiments. T/S, telomeres/single copy gene. *** *p* < 0.001.

Compared with the control, HPAECs in the PI group presented with longer TL (Figure 7A), although PI hypoxia exposure caused a significant accumulation of senescent and apoptotic cells. Smad3-OE and CCG-203971 group versus the PI group had relatively shorter TL (Figure 7A). Generally, TL was found to be positively associated with HPAECs senescence evaluated via the density of SA-β-gal staining (R^2^ = 0.527, *p* < 0.001) and apoptosis (R^2^ = 0.416, *p* < 0.001) evaluated via flow cytometry (Figure 7B,C).

**Figure 8:**
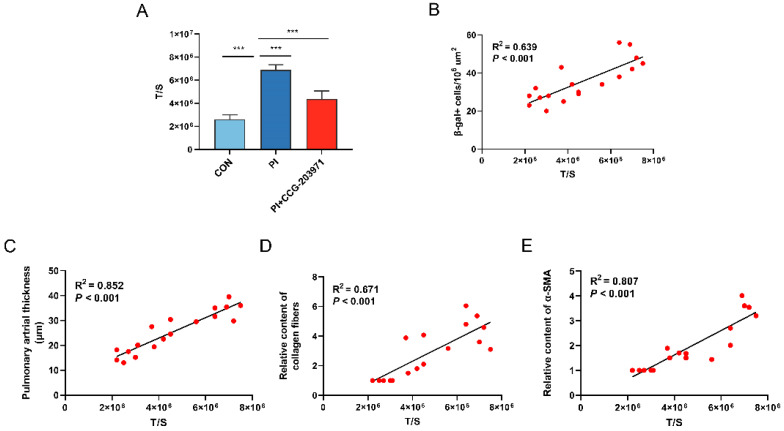
TL is prolonged and associated with senescence and pulmonary artery remodeling after PI hypoxia exposure in vivo. (**A**) TL evaluated as the ratio of T/S was calculated according to the results of Western Blot in lung tissue of rats. (**B**) Correlation analysis of T/S ratio with the abundance of SA β-Gal+ cells in lung tissue. Further correlation analysis of the T/S ratio with the (**C**) thickness of pulmonary arterial vessels, (**D**) level of collagen fiber and (**E**) α-SMA expression in lung tissue. Data presented are mean ± SD of three independent experiments. T/S, telomeres/single copy gene. *** *p* < 0.001.

Consistent with the in vitro study, lung tissue cells collected from the PI group exhibited longer TL than those from the control (Figure 8A). PI + CCG-203971 group demonstrated significantly shorter TL compared with the PI group. The positive correlation of TL with cellular senescence was also verified in vivo studies (R^2^ = 0.639, *p* < 0.001) (Figure 8B). TL was positively associated with the remodeling indices of pulmonary arterial thickness (R^2^ = 0.852, *p* < 0.001), collagen fiber amount (R^2^ = 0.6713, *p* < 0.001,) and α-SMA expression (R^2^ = 0.807, *p* < 0.001) (Figure 8C–E).

## 4. Discussion

**Figure 9:**
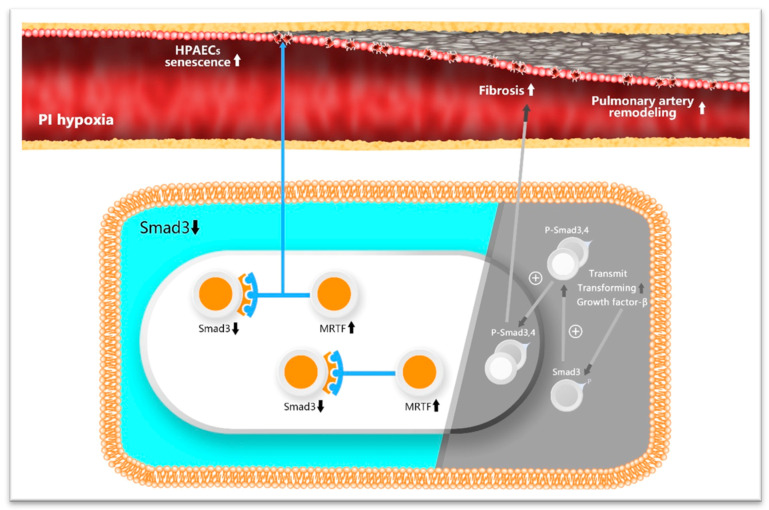
Schematics of PI hypoxia-induced HPAECs senescence mediated via an imbalance of Smad3/MRTF in the formation of pulmonary arterial remodeling. On the left (in bright color): PI hypoxia causes an imbalance of Smad3/MRTF, specifically Smad3 loss and MRTF overexpression, leading to senescence of HPAECs senescence and pulmonary arterial remodeling. On the right (in gray color): PI hypoxia activates canonical phosphorylation of Smad3. The P-Smad3,4 complex enters the nucleus and exerts a fibrogenic effect.

Our study shows that PI hypoxia causes an imbalance of Smad3/MRTF, specifically Smad3 loss and MRTF overexpression, which leads to the senescence of HPAECs and is closely associated with pulmonary arterial remodeling (Figure 9). Treatment targeting the Smad3/MRTF imbalance by increasing Smad3 expression or inhibiting MRTF has the potential to alleviate the senescence of HPAECs and the severity of remodeling. This study provides insight into the treatment of OS-associated pulmonary arterial diseases.

Animal studies have shown that both IH with a trough FiO_2_ of 4% for 6 w [24] and chronic hypoxia with a FiO_2_ of 10% for 4 w [16] can cause pulmonary arterial remodeling and pulmonary hypertension. To the best of our knowledge, this is the first study to demonstrate that a shorter duration of hypoxia, involving only 3 w of PI hypoxia with a trough FiO_2_ of 8%, can lead to significant pulmonary arterial remodeling as evidenced by anatomopathological changes. This finding partially explains why patients with moderate-to-severe OS have a higher prevalence of pulmonary hypertension compared to those with isolated COPD or OSA. This is because they may experience further nocturnal desaturation on top of diurnal hypoxia.

MRTF, which drives the α-SMA promoter and mediates hyperplasia and fibrosis, has been shown to form a stable complex with the Smad3 protein in the nucleus by binding to its MH2 region [25,26]. Physiologically, the intracellular environment keeps a stable concentration of Smad3, which maintains a proportionally balanced relationship with MRTF [23]. Under normal conditions, Smad3 expression prevents excessive MRTF activation and pathological processes. But in stressful conditions, harmful stimulation can lead to the disruption of the Smad3-MRTF interaction, resulting in reduced levels of Smad3 and the consequent loss of its ability to inhibit MRTF [13]. To date, there have been few studies investigating the role of Smad3/MRTF imbalance in the pathogenesis of pulmonary vascular diseases. Our study unveiled the imbalance of Smad3/MRTF induced by PI hypoxia and linked it with the genesis of pulmonary arterial remodeling. Our findings are consistent with those of Zabini et al. [13], who observed that silencing Smad3 expression led to excessive activation of MRTF, resulting in increased expression of α-SMA and actin stress fibers in cultured pulmonary artery smooth muscle cells; these unfavorable effects were reversed via the stimulation of Smad3 expression. It is worth noting that the activation of MRTF in conjunction with the loss of Smad3 implies that the interaction between Smad3 and MRTF may be altered during PI hypoxia. In other words, MRTF activation even might be independent of Smad3 expression in hypoxia. Further investigations are also necessary to determine the cellular localization of MRTF, particularly under pathological conditions.

Our study has revealed that senescence, along with other pathological processes such as apoptosis and fibrosis, plays a significant role in the development of pulmonary arterial remodeling induced by PI hypoxia. This result is consistent with the recent evidence identifying a direct causal role for endothelial senescence in pulmonary hypertension [27,28]. To the best of our knowledge, our study has revealed for the first time that PI hypoxia-induced senescence in vivo and in vitro is mediated via Smad3/MRTF imbalance. Primarily, normal expression of Smad3 is a fundamental protector against senescence, as also shown by the findings of Yang and colleagues who observed that inhibition of Smad3 led to premature senescence-like phenotype in human umbilical vein endothelial cells [29], whereas the inhibition of endogenous miR-216a attenuated the senescent status via promotion of Smad3 [30]. Additionally, we found that MRTF activation is associated with HPAECs senescence, and that CCG-203971, an MRTF inhibitor, attenuated senescence in vivo and prevented hypoxia-associated pulmonary arterial remodeling via MRTF inhibition and subsequent Smad3 promotion. MRTF antagonism using CCG-203971 represents a promising novel target for preventing senescence and pulmonary arterial remodeling. This is particularly significant given the concerns regarding the tumorigenic potential of artificially overexpressed Smad3. By inhibiting MRTF activity, CCG-203971 may offer a safer and more effective approach to attenuating senescence and pulmonary arterial remodeling. Further research is needed to evaluate the anti-senescent competency of other modalities that maintain the balance of Smad3/MRTF in pathological environments.

Telomeres are DNA-protein complexes located at the end of chromosomes that protect the chromosome terminals from fusion and degradation. TL is commonly used to evaluate senescence since the shortening of chromosomal telomeres occurs at the cellular division [31]. However, studies on the association of TL with OSA prevalence are limited and inconsistent. For example, in Judith’s study, patients with severe OSA (AHI > 30 events/h) had greater leukocyte telomere attrition [32], whereas our previous studies did not find a shortening of leukocyte TL in patients with OSA nor a relationship of TL with the cardiovascular prognosis [8]. The inconsistencies between TL and senescence extend to other populations/stress inducers as well and cellular senescence is not always associated with telomere shortening [33]. In the current experiment, both cultured HPAECs and lung tissue exposed to chronic hypoxia exhibited an unexpected increase in TL, despite a high expression of the specific senescence markers. This phenomenon is consistent with the results of previous studies on human umbilical vein endothelial cells exposed to consistent hypoxia (FiO_2_ 10%) [34]. Further, a prior study examining the TL in the small epithelial cells in the lung of COPD patients also showed increased senescence biomarkers (p16) without TL attrition [35]. It is also likely that the telomere lengthening in our study may be an initial adaptive response to hypoxia-related stress, which becomes maladaptive as it exposes the cell to further DNA damage with continued exposure to hypoxic stress [33]. Further research could be conducted to investigate the correlation between Smad/MRTF and telomerase activity, DNA damage and senescence in various cell types.

We acknowledge that our study has some limitations. First, although we found that the overexpression of Smad3 significantly reduced senescence in HPAECs, we did not directly verify this strategy in vivo. Instead, we discovered that inhibiting MRTF, a protein that opposes Smad3, can partially restore Smad3/MRTF balance and have an anti-senescent effect both in vitro and in vivo. Second, we recognize that exposing cells and animals to hypoxia for 72 h and 21 d, respectively, may not fully replicate the chronic oxygen deprivation experienced by OS patients. Third, our study primarily focused on the role of HPAEC senescence in the development of pulmonary arterial remodeling, which is a complex process involving multiple factors. Further research is needed to explore the interplay between senescence and other pathological reactions, such as apoptosis, fibrosis, and overproliferation in the development of pulmonary arterial diseases. Therefore, it may be prudent to perform biopsies on appropriate OS patients to better understand the paradox between senescence and prolonged TL in OSA (hypoxia).

## 5. Conclusions

In summary, our findings from cytological and animal models suggest that an imbalance between Smad3 and MRTF, specifically the loss of Smad3 and activation of MRTF, plays a role in PI hypoxia-induced senescence and pulmonary arterial remodeling. Our in vivo experiments have demonstrated that MRTF inhibition results in a reduction in senescence and anatomical improvement of pulmonary arteries. This effect is partly attributed to a decrease in the severity of senescence. These findings suggest that MRTF antagonism probably has potential as a standalone or combination therapy for pulmonary arterial diseases. The overexpression of Smad3 has shown the potential in restoring the balance between Smad3 and MRTF, leading to a reduction in senescence in vitro. However, further studies are needed to verify its anti-senescent and anti-remodeling effects in vivo, e.g., via MRTF antagonism, while ensuring safety. Further investigation into this approach could provide critical information regarding its potential as a therapeutic strategy for pulmonary hypertension.

## Data Availability

The data that support the findings of this study are available from the corresponding author, J.X., upon request.

## Supplementary Materials

The following supporting information can be downloaded at: https://www.mdpi.com/article/10.3390/biomedicines11092351/s1, Figure S1: The efficiency of Smad3 overexpression through lentiviral transfection. Data presented are mean ± SD of three independent experiments; Table S1: Primer sequences in the experiments.

## References

[1] Shawon M.S., Perret J.L., Senaratna C.V., Lodge C., Hamilton G.S., Dharmage S.C. (2017). Current evidence on prevalence and clinical outcomes of co-morbid obstructive sleep apnea and chronic obstructive pulmonary disease: A systematic review. Sleep Med. Rev..

[2] Soler X., Gaio E., Powell F.L., Ramsdell J.W., Loredo J.S., Malhotra A., Ries A.L. (2015). High Prevalence of Obstructive Sleep Apnea in Patients with Moderate to Severe Chronic Obstructive Pulmonary Disease. Ann. Am. Thorac. Soc..

[3] Xie J., Li F., Wu X., Hou W. (2019). Prevalence of pulmonary embolism in patients with obstructive sleep apnea and chronic obstructive pulmonary disease: The overlap syndrome. Heart Lung.

[4] Adir Y., Humbert M., Chaouat A. (2021). Sleep-related breathing disorders and pulmonary hypertension. Eur. Respir. J..

[5] Ramirez R., Ceprian N., Figuer A., Valera G., Bodega G., Alique M., Carracedo J. (2022). Endothelial Senescence and the Chronic Vascular Diseases: Challenges and Therapeutic Opportunities in Atherosclerosis. J. Pers. Med..

[6] Ramadhiani R., Ikeda K., Miyagawa K., Ryanto G.R.T., Tamada N., Suzuki Y., Kirita Y., Matoba S., Hirata K.I., Emoto N. (2023). Endothelial cell senescence exacerbates pulmonary hypertension by inducing juxtacrine Notch signaling in smooth muscle cells. iScience.

[7] Born E., Lipskaia L., Breau M., Houssaini A., Beaulieu D., Marcos E., Pierre R., Do Cruzeiro M., Lefevre M., Derumeaux G. (2023). Eliminating Senescent Cells Can Promote Pulmonary Hypertension Development and Progression. Circulation.

[8] Polonis K., Becari C., Chahal C.A., Zhang Y., Allen A.M., Kellogg T.A., Somers V.K., Singh P. (2020). Chronic Intermittent Hypoxia Triggers a Senescence-like Phenotype in Human White Preadipocytes. Sci. Rep..

[9] Xing J., Ying Y., Mao C., Liu Y., Wang T., Zhao Q., Zhang X., Yan F., Zhang H. (2018). Hypoxia induces senescence of bone marrow mesenchymal stem cells via altered gut microbiota. Nat. Commun..

[10] Yoon J.H., Sudo K., Kuroda M., Kato M., Lee I.K., Han J.S., Nakae S., Imamura T., Kim J., Ju J.H. (2015). Phosphorylation status determines the opposing functions of Smad2/Smad3 as STAT3 cofactors in T_H_17 differentiation. Nat. Commun..

[11] Gong J., Zhou D., Jiang L., Qiu P., Milewicz D.M., Chen Y.E., Yang B. (2020). In Vitro Lineage-Specific Differentiation of Vascular Smooth Muscle Cells in Response to SMAD3 Deficiency: Implications for SMAD3-Related Thoracic Aortic Aneurysm. Arterioscler. Thromb. Vasc. Biol..

[12] Chen B., Huang S., Su Y., Wu Y.J., Hanna A., Brickshawana A., Graff J., Frangogiannis N.G. (2019). Macrophage Smad3 Protects the Infarcted Heart, Stimulating Phagocytosis and Regulating Inflammation. Circ. Res..

[13] Zabini D., Granton E., Hu Y., Miranda M.Z., Weichelt U., Breuils Bonnet S., Bonnet S., Morrell N.W., Connelly K.A., Provencher S. (2018). Loss of SMAD3 Promotes Vascular Remodeling in Pulmonary Arterial Hypertension via MRTF Disinhibition. Am. J. Respir. Crit. Care Med..

[14] Cheng P., Wirka R.C., Kim J.B., Kim H.J., Nguyen T., Kundu R., Zhao Q., Sharma D., Pedroza A., Nagao M. (2022). Smad3 regulates smooth muscle cell fate and mediates adverse remodeling and calcification of the atherosclerotic plaque. Nat. Cardiovasc. Res..

[15] Song J.Q., Jiang L.Y., Fu C.P., Wu X., Liu Z.L., Xie L., Wu X.D., Hao S.Y., Li S.Q. (2020). Heterozygous SOD2 deletion deteriorated chronic intermittent hypoxia-induced lung inflammation and vascular remodeling through mtROS-NLRP3 signaling pathway. Acta Pharmacol. Sin..

[16] Ball M.K., Waypa G.B., Mungai P.T., Nielsen J.M., Czech L., Dudley V.J., Beussink L., Dettman R.W., Berkelhamer S.K., Steinhorn R.H. (2014). Regulation of hypoxia-induced pulmonary hypertension by vascular smooth muscle hypoxia-inducible factor-1alpha. Am. J. Respir. Crit. Care Med..

[17] Zhao X., Zhu D.M., Gan W.J., Li Z., Zhang J.L., Zhao H., Zhou J., Li D.C. (2013). Lentivirus-mediated shRNA interference targeting vascular endothelial growth factor inhibits angiogenesis and progression of human pancreatic carcinoma. Oncol. Rep..

[18] Valieva Y., Ivanova E., Fayzullin A., Kurkov A., Igrunkova A. (2022). Senescence-Associated beta-Galactosidase Detection in Pathology. Diagnostics.

[19] Rouault C., Marcelin G., Adriouch S., Rose C., Genser L., Ambrosini M., Bichet J.C., Zhang Y., Marquet F., Aron-Wisnewsky J. (2021). Senescence-associated beta-galactosidase in subcutaneous adipose tissue associates with altered glycaemic status and truncal fat in severe obesity. Diabetologia.

[20] Dungan C.M., Murach K.A., Zdunek C.J., Tang Z.J., Nolt G.L., Brightwell C.R., Hettinger Z., Englund D.A., Liu Z., Fry C.S. (2022). Deletion of SA beta-Gal+ cells using senolytics improves muscle regeneration in old mice. Aging Cell.

[21] Schindelin J., Rueden C.T., Hiner M.C., Eliceiri K.W. (2015). The ImageJ ecosystem: An open platform for biomedical image analysis. Mol. Reprod. Dev..

[22] Tian H., Liu L., Wu Y., Wang R., Jiang Y., Hu R., Zhu L., Li L., Fang Y., Yang C. (2021). Resistin-like molecule beta acts as a mitogenic factor in hypoxic pulmonary hypertension via the Ca^2+^-dependent PI3K/Akt/mTOR and PKC/MAPK signaling pathways. Respir. Res..

[23] Charbonney E., Speight P., Masszi A., Nakano H., Kapus A. (2011). beta-catenin and Smad3 regulate the activity and stability of myocardin-related transcription factor during epithelial-myofibroblast transition. Mol. Biol. Cell.

[24] Hao S., Jiang L., Fu C., Wu X., Liu Z., Song J., Lu H., Wu X., Li S. (2019). 2-Methoxyestradiol attenuates chronic-intermittent-hypoxia-induced pulmonary hypertension through regulating microRNA-223. J. Cell. Physiol..

[25] Masszi A., Speight P., Charbonney E., Lodyga M., Nakano H., Szaszi K., Kapus A. (2010). Fate-determining mechanisms in epithelial-myofibroblast transition: Major inhibitory role for Smad3. J. Cell Biol..

[26] Hua F., Zhou J., Liu J., Zhu C., Cui B., Lin H., Liu Y., Jin W., Yang H., Hu Z. (2010). Glycogen synthase kinase-3beta negatively regulates TGF-beta1 and Angiotensin II-mediated cellular activity through interaction with Smad3. Eur. J. Pharmacol..

[27] van der Feen D.E., Bossers G.P.L., Hagdorn Q.A.J., Moonen J.R., Kurakula K., Szulcek R., Chappell J., Vallania F., Donato M., Kok K. (2020). Cellular senescence impairs the reversibility of pulmonary arterial hypertension. Sci. Transl. Med..

[28] Culley M.K., Chan S.Y. (2022). Endothelial Senescence: A New Age in Pulmonary Hypertension. Circ. Res..

[29] Yang S., Mi X., Chen Y., Feng C., Hou Z., Hui R., Zhang W. (2018). MicroRNA-216a induces endothelial senescence and inflammation via Smad3/IkappaBalpha pathway. J. Cell Mol. Med..

[30] Chen Y., Wang S., Yang S., Li R., Yang Y., Chen Y., Zhang W. (2021). Inhibitory role of ginsenoside Rb2 in endothelial senescence and inflammation mediated by microRNA-216a. Mol. Med. Rep..

[31] Zhu Y., Liu X., Ding X., Wang F., Geng X. (2019). Telomere and its role in the aging pathways: Telomere shortening, cell senescence and mitochondria dysfunction. Biogerontology.

[32] Carroll J.E., Irwin M.R., Seeman T.E., Diez-Roux A.V., Prather A.A., Olmstead R., Epel E., Lin J., Redline S. (2019). Obstructive sleep apnea, nighttime arousals, and leukocyte telomere length: The Multi-Ethnic Study of Atherosclerosis. Sleep.

[33] Victorelli S., Passos J.F. (2017). Telomeres and Cell Senescence—Size Matters Not. EBioMedicine.

[34] Guan J.Z., Guan W.P., Maeda T., Makino N. (2012). Different levels of hypoxia regulate telomere length and telomerase activity. Aging Clin. Exp. Res..

[35] Birch J., Anderson R.K., Correia-Melo C., Jurk D., Hewitt G., Marques F.M., Green N.J., Moisey E., Birrell M.A., Belvisi M.G. (2015). DNA damage response at telomeres contributes to lung aging and chronic obstructive pulmonary disease. Am. J. Physiol. Lung Cell Mol. Physiol..

