# Supplementary material for: Persistent Hypoxia with Intermittent Aggravation Causes Imbalance in Smad3/Myocardin-Related Transcription Factor Signaling with Consequent Endothelial Senescence and Pulmonary Arterial Remodeling
Source: Biomedicines. 2023 Aug 23;11(9):2351. doi: 10.3390/biomedicines11092351 (PMC10526072; doi:10.3390/biomedicines11092351)
Supplement: Supplementary file 1 [file biomedicines-11-02351-s001.zip › Supplementary File S1.pdf]

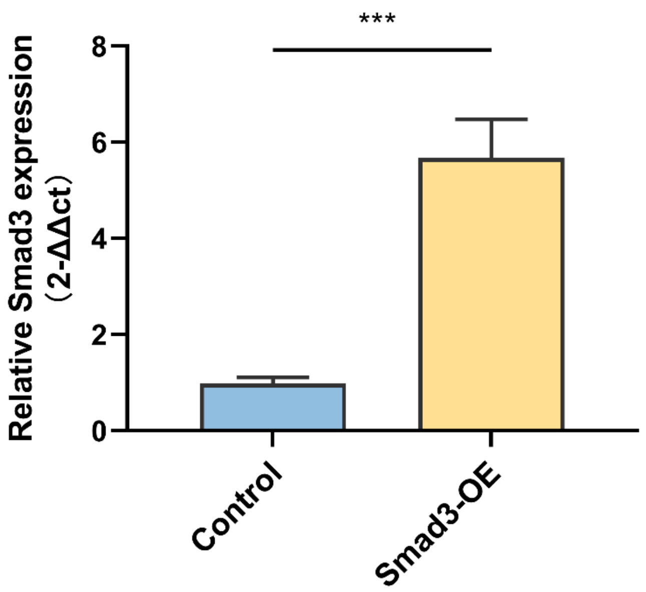

Figure S1. The efficiency of Smad3 overexpression through lentiviral transfection. Data presented are mean ± SD of three independent experiments. ***$p < 0.001$.
