# Supplementary material for: Persistent Hypoxia with Intermittent Aggravation Causes Imbalance in Smad3/Myocardin-Related Transcription Factor Signaling with Consequent Endothelial Senescence and Pulmonary Arterial Remodeling
Source: Biomedicines. 2023 Aug 23;11(9):2351. doi: 10.3390/biomedicines11092351 (PMC10526072; doi:10.3390/biomedicines11092351)
Supplement: Supplementary file 1 [file biomedicines-11-02351-s001.zip › Supplementary File S2.pdf]

Supplemental Methods

qRT-PCR

Total RNAs from cells or animals were isolated using TRIzol reagent (Invitrogen, USA) as per manufacturer's instructions. Nanodrop spectrophotometer (Thermo Fisher Scientific, USA) was used to detect the concentration of RNA. cDNA was synthesized using HiScript III All-in-one RT SuperMix (Vazyme, China) following the manufacturer's instructions. qRT-PCR was performed with AceQ qPCR SYBR Green Master Mix (Vazyme, China) to detect mRNA levels. The primer sequences are shown in Table 1. The relative mRNA level to the control GAPDH (in vivo) or β-actin (in vitro) was calculated using the $2^{\wedge(-\Delta\Delta Ct)}$ method. The relative telomere/36B4 (T/S) ratio of each sample was calculated to reflect the relative TL.

Table S1. Primer sequences in the experiments.

Gene	Primer sequence (5'-3')
Human-Smad3	F:CTGGGGTTAGGTCACTGCTG
	R: CTGTGGGAATGTCGCATCCT
Human-Telomere	F： ACACTAAGGTTTGGGTTTGGGTTTGGGTTTGGGTTAGTGT
	R： TGTTAGGTATCCCTATCCCTATCCCTATCCCTATCCCTAACA
Rats-Telomere	F: CGGTTTGTTGGGTTTGGGTTTGGGTTTTGGGTTTGGGTT
	R: GGCTTGCCTTTACCCTTACCCTTACCCTTACCCTTACCCT
Human-36B4	F: ATGCAGCAGATCCGCATGT
	R: TTGCGCATCATGGTGTTCTT
Rats-36B4	F: ACTGGTCTAGGACCCGAGAAG

	R: TCAATGGTGCCTCTGGAGATT
Human-$p16^{INK4A}$	F: GGGGTCGGGTAGAGGAGG
	R: GCCCATCATCATGACCTGGA
Rats- $p16^{INK4A}$	F: TGAATCTCCGCGAGGAAAGC
	R： TGCCCATCATCATCACCTGAA
Human-β-actin	F: GCTATGTTGCCCTAGACTTCGA
	R: GATGCCACAGGATTCCATACC
Rats-GAPDH	F: TGACTTCAACAGCGACACCCA
	R: CACCCTGTTGCTGTAGCCAAA

Western blot

Total protein from HPAECs and lung tissue were extracted using RIPA buffer (Beyotime, China) mixed with 1% Protease Inhibitor (Beyotime, China). Equal amount of total protein (30 μg) was separated by 10% SDS-PAGE (Epizyme, China) and transferred onto the polyvinylidene fluoride membrane (PVDF) membrane (Millipore, USA). Then, the membranes were blocked with Protein Free Rapid Blocking Buffer (Epizyme, China) for 30 min. After that, the membranes were incubated with primary antibodies for β-Actin (1:5000; 66009-1-Ig, Proteintech, US), MRTF (1:1000; ab115319, Abcam, UK), and Smad3 (1:1000; ab40854, Abcam, UK) overnight at 4℃ and followed by washing and incubation with secondary HRP-labeled antibodies: goat anti-rabbit antibody (1:3000; A0208, Beyotime, China), goat anti-mouse antibody (1:3000; A0216, Beyotime, China) at room temperature for 1 h. Protein specific bands were detected through immobilon Western Chemiluminescent HRP Substrate kit (Millipore, USA), and visualized chemiluminescence imager (GE, USA). Relative protein levels were calculated by Image J Software. The Smad3/MRTF ratio was calculated by dividing the protein level of Smad3 by the protein level of MRTF as

determined by the WB. The relationship between the SMAD3/MRTF ratio and other variables were then examined by Pearsons's correlation analysis.

Cell viability assay

HPAECs were seeded into 96-well plate (1×10^3 cells/well), and were subjected to normoxia or PI hypoxia treatment at 37°C for 72 h. The proliferation activity of HPAECs was detected according to the instructions of the CCK-8 kit (College of Japan, Japan). CCK-8 solution of 10 μL was added into each well of HPAECs cultured and incubated at 37°C for 2 h. The absorbance was measured at 450 nm using a microplate reader (Tecan, Switzerland) to calculate cell proliferative viability.

Enzyme-linked immunosorbent assay (ELISA)

ELISA assay was conducted to evaluate the concentration of senescence-associated secretory phenotypes (SASP) factor of IL-1, TNF-α, and MCP1 (Sigma-Aldrich, USA). Briefly, the supernatants of 1×10^5 HPAECs or 300-500 ul peripheral blood from rats were collected at the indicated time points, then the fluid and reagents were added to 96-well plates according to the instructions. The absorbance of each microwell at 450 nm was detected and the concentration was calculated according to the standard curve.

Flow cytometery assay for cell apoptosis

The Annexin V-APC/PI apoptosis detection kit (Southern Biotech, USA) was used to assess the cellular apoptosis according to the manufacturer's instructions. HPAECs were suspended by trypsinization, centrifuged at 1,300 rpm for 3 min and washed with PBS. The cells were suspended in annexin V binding buffer. APC-labeled Annexin V and propidium iodide were added to cells followed by 15 min incubation in the dark. The mixture was centrifuged at 1,500 rpm for 3 min to remove the supernatant and then 1000 μL diluted 1 × binding buffer was added. Cells were tested by MM high-pass flow cytometer (Millipore, Massachusetts, USA), with percentage of apoptotic cells analyzed by FlowJo software (Treestar, USA).
